# Effect of local anesthetic concentration, dose and volume on the duration of single-injection ultrasound-guided axillary brachial plexus block with mepivacaine: a randomized controlled trial

**DOI:** 10.1186/s12871-015-0110-0

**Published:** 2015-09-30

**Authors:** Maaike G. E. Fenten, Karin P. W. Schoenmakers, Petra J. C. Heesterbeek, Gert Jan Scheffer, Rudolf Stienstra

**Affiliations:** Department of Anesthesiology, Sint Maartenskliniek, Postbox 9011, 6500 GM Nijmegen, The Netherlands; Research Department, Sint Maartenskliniek, Nijmegen, The Netherlands; Department of Anesthesiology, Radboud University Medical Center, Nijmegen, The Netherlands

**Keywords:** Axillary brachial plexus block, Mepivacaine, Volume, Concentration, Dose

## Abstract

**Background:**

In what way volume, concentration and dose affect block duration is controversial. The purpose of the present study is to investigate the effect of dose, volume and concentration of mepivacaine on the duration of sensory and motor blockade in ultrasound-guided single shot axillary brachial plexus blockade.

**Methods:**

In this parallel group randomized trial conducted in the Sint Maartenskliniek Nijmegen, 45 adult patients undergoing minor orthopaedic forearm, wrist or hand surgery were randomized to 3 groups. Group A: 20 mL mepivacaine 1.5 %, Group B: 30 mL mepivacaine 1 % and Group C: 30 mL mepivacaine 1.5 %. Randomization was computer-generated, with allocation concealment by opaque sequentially numbered sealed envelopes. Patients and observers were blinded to group allocation. Primary outcome measure: duration of sensory block.

**Results:**

Forty-five patients were randomized, four patients were excluded and replaced, and 15 patients in each group were included in the analysis. Mean (95 % CI) sensory and motor block duration was 256 (230–282) and 254 (226–282) minutes in Group A, 226 (209–243) and 220 (200–240) minutes in Group B and 270 (249–291) and 264 (244–284) minutes in Group C. Duration of sensory and motor block duration differed significantly between groups (*p* = 0.012 and *p* = 0.016 respectively). Post-hoc analysis showed a significantly reduced sensory and motor block duration in Group B when compared to Group C of 44 min. No local anesthetic systemic toxicity was reported.

**Conclusions:**

When using mepivacaine for axillary brachial plexus block, a higher dose and concentration was associated with a longer duration of sensory and motor blockade, but not a higher volume.

**Trial Registration:**

The Netherlands National Trial Register NTR3648. Registered October 3, 2012.

## Background

The introduction of ultrasound has changed the practice of peripheral nerve block (PNB). Using ultrasound guidance, local anesthetic (LA) spread around the nerves can be assessed with the possibility of repositioning the needle in case of maldistribution, [1] allowing for a reduction in LA dose without compromising the quality of PNB. Recent publications indeed illustrate that the volume of LA can be significantly reduced when particular regional anesthetic techniques are performed with ultrasound guidance [2–5]. While dose reduction is advantageous from a safety perspective, an unwanted tradeoff may be a shorter duration of the nerve blockade.

One of the factors affecting the duration of peripheral nerve block is the dose of LA, dose being the product of volume and concentration. In what way volume, concentration and dose of LA affect block duration is subject to debate [6]. In a recent study, we compared the duration of sensory and motor block of 15 and 40 mL mepivacaine 1.5 % for axillary brachial plexus blockade (ABPB) using ultrasound guidance [7]. Volume reduction from 40 mL to 15 mL (62.5 %) shortened the overall duration of sensory and motor block by approximately 17–19 %, reduced sensory and motor block duration of individual nerves by 18–40 % and decreased the time to first request of postoperative analgesia by approximately 30 %. The difference in block duration observed in this study was the effect of either a reduction in volume from 40 to 15 mL or a reduction in dose from 600 to 225 mg. We designed the present study to determine if the reduction in duration of sensory and motor blockade in APBP is mainly affected by volume reduction or by dose reduction of mepivacaine. The null hypothesis was that sensory block duration is not affected by dose and volume reduction.

## Methods

This study was set up as a Phase IV, monocenter, double-blinded (for observer and patient), parallel group randomized (1:1:1) trial. No protocol amendments were made during the study conduct. The study was approved by the Independent Review Board Nijmegen and was registered with the Nederlands Trial Register (www.trialregister.nl, number NTR3648) before onset of participant enrollment.

Eligible patients were all adults aged 18 or over with ASA physical health classification I–III, scheduled for single-injection ABPB for hand, wrist or forearm surgery. Exclusion criteria included contra-indications for regional anesthesia (infection at the injection site, coagulopathy), known hypersensitivity to amide-type local anesthetics, and known history of peripheral neuropathy. Specific criteria for withdrawal and replacement included: failure to perform adequate single-injection ABPB and failure to complete the study protocol. Patients were assessed for eligibility during the preoperative screening visit. Patients were informed about the study verbally and in writing and written informed consent was obtained from all patients.

The study was conducted at the Sint Maartenskliniek Nijmegen, The Netherlands between October 2012 and June 2014 according to the Declaration of Helsinki and later revisions thereof and in accordance with the ICH guidelines for Good Clinical Practice. The Sint Maartenskliniek specializes in posture and movement. The orthopedic center is facilitated by an anesthesiology department specialized in locoregional and regional anesthesia techniques.

### Study procedure

Study medication was prepared by an anesthetic nurse not involved in the study and was disclosed to the anesthesiologist performing the block procedure. Study medication consisted of either 20 mL mepivacaine 1.5 %; 300 mg (Group A), 30 mL mepivacaine 1.0 %; 300 mg (Group B), or 30 mL mepivacaine 1.5 %; 450 mg (Group C).

After establishing intravenous access and routine monitoring (ECG, non-invasive blood pressure and peripheral oxygen saturation), ABPB was performed under ultrasound guidance using a short axis, in-plane technique. All blocks were placed by experienced anesthesiologists with the assistance of an anesthetic nurse. Blocks were performed under aseptic conditions using chlorhexidine skin preparation and sterile ultrasound probe covers.

The patient was placed in the supine position with the head facing away from the arm to be blocked, the arm abducted and the elbow flexed in 90°. A 100-mm 22-gauge insulated short bevel needle (Stimuplex®; B. Braun, Melsungen, Germany) was inserted laterally in the axilla under ultrasound guidance. The musculocutaneous, median, ulnar and radial nerve were identified using ultrasound and the tip of the needle was brought in proximity of each individual nerve subsequently. The needle was connected to a nerve stimulator (Stimuplex® HNS 11; B. Braun) set to deliver 100 nC (0.1 ms, 1 mA) in order to facilitate identification of the individual nerves. The nerves were identified and blocked separately with one fourth of the study medication per nerve. Per patient one skin puncture was made, the needle was retracted subcutaneously and redirected under ultrasound guidance to approach the nerves individually. Time was designated t = 0 upon conclusion of the block procedure.

In case of insufficient analgesia at the surgical site at t = 30 min, an additional rescue block was placed in the block room, or surgery was performed under general anesthesia. These patients were excluded from further analysis and replaced.

Surgery was performed under regional anesthesia. In case of patient discomfort or upon patient request, sedation was provided with propofol (25–60 μg.kg^−1^.min^−1^) and remifentanil (0.01–0.04 μg.kg^−1^.min^−1^).

The patients received paracetamol 1 g orally four times daily and etoricoxib 90 mg orally once a day, starting on the morning of surgery. When the block started to wear off, additional postoperative pain treatment consisted of morphine 0.1-0.15 mg/kg every 4 h subcutaneously upon patient request.

### Primary and secondary outcome measures

The primary outcome parameter was duration of sensory block. Secondary outcome parameters included duration of motor block, duration of sensory and motor block of individual nerves, block onset time, time to first request for additional postoperative pain treatment (TTFR) and patient satisfaction (NRS 0–10) with the anesthetic technique.

After injection of the local anesthetic solution, the onset of sensory and motor block was assessed every 5 min, until 30 min after injection. Sensory block of the medial antebrachial cutaneous, musculocutaneous, radial, median and ulnar nerves was assessed by pinprick. Sensory block was scored on a three-point scale as 0 = absent, 1 = partial and 2 = complete. At the same intervals, motor block of the musculocutaneous, radial, median and ulnar nerve was assessed (see Table 1) on a similar three-point scale (0 = no, 1 = partial and 2 = complete motor block). A complete overall sensory block was defined as a total score of 10; complete overall motor block was defined as a total score of 8.

**Table 1:** Baseline characteristics

	Group A (*n* = 15)	Group B (*n* = 15)	Group C (*n* = 15)
Sex, no. M/ no. F	2/13	6/9	7/8
Age (yr)	59 ± 9	49 ± 13	53 ± 15
Height (cm)	165 ± 7	172 ± 8	171 ± 11
Weight (kg)	71 ± 13	75 ± 7	78 ± 17
ASA classification, no. 1/no. 2/ no. 3	3/10/2	10/5/0	7/8/0
Duration of surgery (min)	24 ± 17	27 ± 25	34 ± 27
Site of surgery, no. left/ no. right	5/10	5/10	5/10
Type of surgery:			
- carpal tunnel release, no.	3	2	4
- trapezoidectomy, no.	6	1	2
- removemal of osteosynthesis material, no.	2	4	1
- arthrodesis of finger, no.	0	2	1
- release trigger finger, no.	2	2	3
- arthrodesis of wrist, no.	0	1	2
- other, no.	2	3	2

Group A: 20 mL mepivacaine 1.5 %. Group B: 30 mL mepivacaine 1.0 %. Group C: 30 mL mepivacaine 1.5 %. Values are absolute numbers, mean ± SD

Upon arrival at the recovery, offset of sensory and motor block was assessed every 15 min in the same manner as preoperatively until full recovery. The primary outcome parameter was overall duration of sensory block defined as the time from t = 0 until the postoperative measurement where total sensory score had returned to zero. Overall duration of motor block was defined as the time from t = 0 until the first postoperative measurement where total motor score had returned to zero. Block onset time was defined as the time from t = 0 until the time sensory respectively motor score was maximal. TTFR was defined as the time interval from t = 0 until the time the first request for postoperative analgesia was made.

### Sample size, randomization and blinding

The sample size calculation was similar to our previous study [7] and based on the overall duration of sensory block. In previous research, we [7], Bugamelli et al. [8], and Robaux et al. [9] found a variation (SD) in duration of sensory peripheral nerve block with mepivacaine of ± 47 min (47, 45 and 43 min respectively). Based on these data, the sample size required to have a 90 % probability of detecting a difference of 60 min (approximately 25 %) in duration of the ABPB between the groups (two sided, level of significance 0.05) is 13 patients per group. Compensating for variations in the standard deviation, we chose to include 15 patients per group. A computer-generated sequence of random numbers in 3 blocks of 15 and a 1:1:1 allocation was used for randomization. The allocation sequence was concealed from the researcher assessing and enrolling patients in sequentially numbered, sealed, opaque envelopes made by an independent researcher, not involved in the study.

A computerized database automatically assigned a study number to each patient assessed for eligibility. Once included in the study, the study number of the patient was written on the sealed randomization envelopes by the researcher. On the day of surgery the envelope was handed over to an anesthetic nurse not involved in the study. The anesthetic nurse prepared the study medication according to the allocated group written on the card inside the envelope, wrote the study number of the patient on the card and resealed the envelope.

Patients with specific withdrawal criteria, as mentioned earlier in the methods section, were excluded and replaced. After 45 patients were included in the study, an independent researcher not involved in the study made additional sealed envelopes for the patients replacing the excluded patients, randomized and sequentially numbered to conceal treatment allocation for the observer.

At the conclusion of the study, all resealed envelopes were checked by an independent researcher not involved in the study, for any violations of the group allocation.

The anesthetic nurse that prepared the study medication was allowed to disclose allocation to the anesthesiologist that performed the block procedure. Both patients and researcher were blinded for the volume and concentration of anesthetic solution used.

### Statistical analysis

Per-protocol analysis was conducted using GraphPad Prism 6 software (GraphPad Software Inc, San Diego, CA).

For statistical comparison between the groups of sensory block (primary outcome parameter), motor block and the duration of individual nerve blocks one-way ANOVA was used and Tukey post-hoc analyses were conducted. Block onset time and patient satisfaction (NRS 0–10) with the anesthetic technique was compared between groups with the Kruskall Wallis test.

For between group comparison on baseline characteristics Chi square test and the Kruskall Wallis test were used. All tests were 2-sided, and a *p*-value ≤ 0.05 was considered statistically significant. Frequency distribution was tested using Kolmogorov-Smirnov test for normality. Data are presented as mean (95 % confidence interval) or median [range] as appropriate.

## Results

**Fig. 1:**
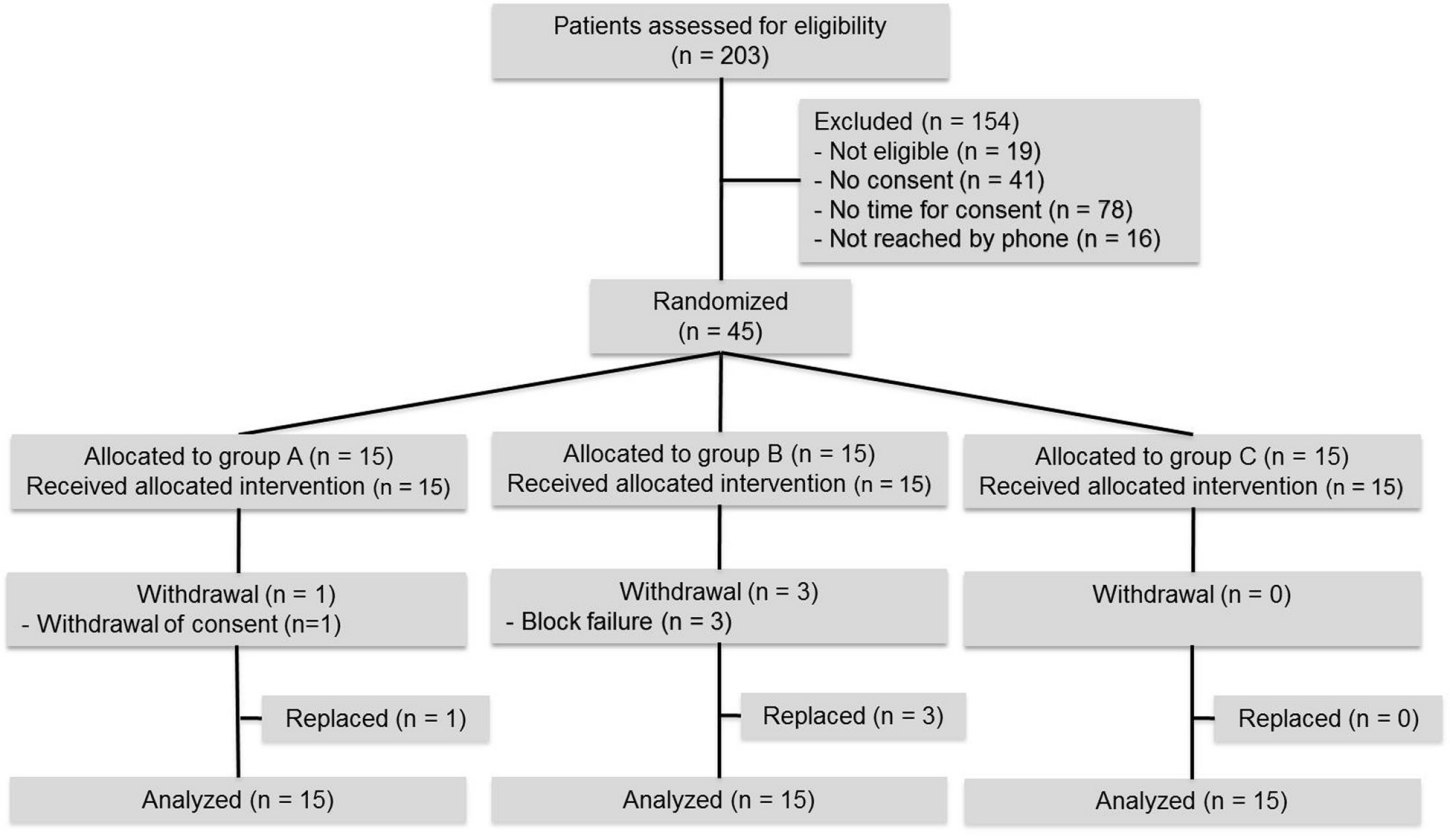
Flowchart of patients enrolled in the study

In total, 45 patients were randomized, 15 patients per group. Four patients were excluded and replaced. Reasons for withdrawal were block failure (three patients in Group B) and patient consent withdrawal (one patient in Group A). All patients received the allocated intervention. A CONSORT flowchart is shown in Fig. 1. Baseline characteristics of the three groups did not differ significantly and are described in Table 1.

Thirty minutes after block placement, a complete sensory block was confirmed in 13 patients in Group A, 7 patients in Group B and 13 patients in Group C (*p* = 0.006). Motor block was complete in 13 patients in Group A, 10 patients in Group B and 14 patients in Group C. Data on sensory and motor block scores of individual nerves after 30 min are shown in Table 2.

**Table 2:** Block scores of individual nerves at 30 minutes

	Group A (*n* = 15)			Group B (*n* = 15)			Group C (*n* = 15)		
Nerve	Score 2	Score 1	Score 0	Score 2	Score 1	Score 0	Score 2	Score 1	Score 0
Med. Anteb. Cut. sens	14	1	–	14	1	–	15	–	–
Musculocutaneous sens	13	2	–	14	1	–	15	–	–
Musculocutaneous mot	13	2	–	12	3	–	14	1	–
Radial sens	14	1	–	10	5	–	14	1	–
Radial mot	14	1	–	12	2	1	13	2	–
Median sens	14	1	–	12	3	–	15	–	–
Median mot	13	2	–	14	1	–	15	–	–
Ulnar sens	14	1	–	13	1	1	14	1	–
Ulnar mot	14	1	–	14	1	–	15	–	–

Group A: 20 mL mepivacaine 1.5 %; Group B: 30 mL mepivacaine 1.0 %; Group C: 30 mL mepivacaine 1.5 %. Med. anteb. cut.: medial antebrachial cutaneous nerve; sens: sensory block score, mot: motor block score. Block was scored on a three-point scale as 0 = absent, 1 = partial and 2 = complete

**Fig. 2:**
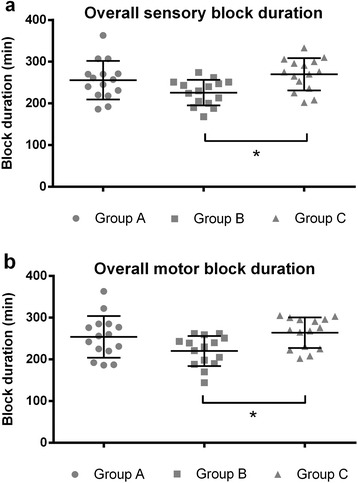
Duration of overall sensory block (**a**) and overall motor block (**b**) per Group. Group A: 20 mL mepivacaine 1.5 %. Group B: 30 mL mepivacaine 1.0 %. Group C: 30 mL mepivacaine1.5 %. Dots represent individual patients, the horizontal lines with error bars represent mean with SD. *p < 0.05

Sensory block, as well as motor block duration, differed significantly between groups: *p* = 0.012 and *p* = 0.016, respectively (Table 3). Post-hoc-between-group analyses showed a statistically shorter sensory and motor block duration of 44 min (20 %) in Group B when compared with Group C. Sensory and motor block duration in group A did not differ significantly from group B and C. Data on between-group differences of sensory and motor block duration are shown in Fig. 2 and Table 4.

**Table 3:** Duration of axillary plexus nerve block

	Group A (*n* = 15)	Group B (*n* = 15)	Group C (*n* = 15)	*p*-value
Block duration (min)				
Overall sensory	256 (230 – 282)	226 (209 – 243)	270 (248 – 291)	**0.012**
Overall motor	254 (226 – 282)	220 (200 – 240)	264 (244 – 284)	**0.016**
Block duration (min)				
Med. Anteb. Cut. nerve				
Sensory	222 (201 – 242)	197 (174–220)	247 (221 – 273)	**0.010**
Musculocutaneous nerve				
Sensory	213 (191 – 235)	196 (175 – 217)	215 (186 – 244)	0.446
Motor	224 (206 – 242)	188 (164 – 212)	216 (197 – 236)	**0.031**
Radial nerve				
Sensory	228 (197 – 259)	207 (190 – 223)	227 (191 – 164)	0.328^a^
Motor	229 (206 – 251)	209 (190 – 229)	258 (238 – 277)	**0.003**
Median nerve				
Sensory	236 (211 – 261)	204 (185 – 223)	248 (224 – 272)	**0.015**
Motor	245 (214 – 275)	200 (175 – 225)	241 (222 – 259)	**0.015** ^b^
Ulnar nerve				
Sensory	238 (211 – 265)	211 (189 – 233)	249 (220 – 278)	0.087
Motor	243 (211 – 274)	209 (185 – 234)	257 (236 – 279)	**0.025**

Group A: 20 mL mepivacaine 1.5 %; Group B: 30 mL mepivacaine 1.0 %; Group C: 30 mL mepivacaïne 1.5 %; med. anteb. cut. nerve: medial antebrachial cutaneous nerve. ^a^no data because of a postoperative cast or bandage in 6 patients in Group A, 5 patients in Group B and 8 patients in Group C. ^b^no data because of a postoperative cast or bandage in 3 patients in group A, 2 patients in Group B and 2 patients in Group C. Values are mean (95 % CI)

Bold data represent statistically significant differences

**Table 4:** Groupwise comparisons of block duration

	Group A vs Group B		Group B vs Group C		Group A vs Group C	
	Difference (95 % CI)	*p*-value	Difference (95 % CI)	*p*-value	Difference (95 % CI)	*p*-value
Block duration (min)						
Overall sensory	30 (−5 – 65)	0.100	**−44 (−79 – -9)**	**0.010**	−14 (−49 – 21)	0.599
Overall motor	34 (−3 – 70)	0.079	**−44 (−80 – -7)**	**0.017**	−10 (−47 – 27)	0.787
Block duration (min)						
Med. Anteb. Cut. nerve						
Sensory	24 (−13 – 62)	0.260	**-50 (−87 – -12)**	**0.007**	−25 (−63 – 12)	0.244
Musculocutaneous nerve						
Sensory	17 (−22 – 56)	0.550	−19 (−58 – 20)	0.482	−2 (−41 – 37)	0.993
Motor	**36 (2 – 69)**	**0.031**	−28 (−61 – 5)	0.110	7 (−26 – 41)	0.848
Radial nerve						
Sensory	22 (−18 – 61)	0.375	−21 (−63 – 22)	0.451	1 (−43 – 44)	0.999
Motor	20 (−13 – 52)	0.320	**−49 (−81 – -16)**	**0.002**	−29 (−62 – 3)	0.086
Median nerve						
Sensory	32 (−4 – 69)	0.091	**−44 (−80 – -8)**	**0.014**	−12 (−48 – 24)	0.709
Motor	**45 (5 – 84)**	**0.024**	**−41 (−79 – -2)**	**0.039**	4 (−35 – 44)	0.963
Ulnar nerve						
Sensory	27 (−15 – 70)	0.272	−38 (−80 – 4)	0.082	−11 (−53 – 32)	0.818
Motor	34 (−8 – 76)	0.137	**−48 (−91 – -6)**	**0.023**	−15 (−57 – 28)	0.684

Group A: 20 mL mepivacaine 1.5 %. Group B: 30 mL mepivacaine 1.0 %. Group C: 30 mL mepivacaine 1.5 %; med. anteb. cut. nerve: medial antebrachial cutaneous nerve; CI: confidence interval. Values are calculated differences (95 % confidence interval of the difference). Multiplicity adjusted *p*-values are given

Bold data represent statistically significant differences

Because of the presence of a postoperative cast, offset of sensory block of the radial nerve could not be evaluated in 19 patients (6, 5 and 8 patients in Group A, B and C respectively). In these patients maximum postoperative sensory block score was 8 and overall sensory block duration was defined as the time until the total sensory score had returned to zero. The offset of motor block of the median nerve could not be tested in 7 patients (3, 2 and 2 patients in Group A, B and C respectively). In these patients maximum postoperative motor block score was 6 and overall motor block duration was defined as the time until the total motor score was returned to zero.

Only seven patients requested additional postoperative pain medication (four in Group A, one in Group B and two in Group C). Because of the limited number of data on TTFR, no average TTFR was calculated. Patient satisfaction with the anesthetic technique (NRS, on a scale 0 – 10) was comparable between Groups; 8.8 ± 0.8 in Group A, 8.7 ± 1.7 in Group B and 8.9 ± 1.1 in Group C (*p* = 0.76).

Twenty-eight patients received sedation upon request during surgery. In all included patients sensory block was adequate, none of the patients requiring conversion to general anesthesia. None of the patients showed signs or symptoms of local anesthetic systemic toxicity during the study procedure. In our hospital all patients are screened for postoperative nerve damage three weeks after surgery. None of the patients enrolled in the study expressed any sign of nerve damage at the postoperative screening.

## Discussion

Because of the inseparable relation between dose, volume and concentration, the issue which of these three entities is the major determinant of duration of nervous blockade is complex. In this study we compared the effects of equal doses in different volume/concentration, as well as different dose/concentration in equal volumes, and different volume/dose in equal concentrations of mepivacaine for ABPB in order to determine whether duration of sensory and motor blockade in APBP is mainly affected by volume, concentration or dose.

Our results show that a higher dose and concentration administered resulted in a longer duration of sensory and motor block. When comparing the groups with equal concentrations in our study, no difference was found in block duration, despite the difference in dose and volume, suggesting a role for concentration and not for dose in determining block duration. When comparing the groups with equal dose, there is a tendency for a longer duration for sensory and motor block in the group with higher concentration and smaller volume. As it is unlikely that a smaller volume would explain this non-significant trend, this may indicate that concentration is proportional to the duration of nerve blockade when using equal doses.

Serradell et al. [10] found no differences in the duration of analgesia when using 36 mL, 28 mL or 20 mL of mepivacaine 1 % for ABPB, suggesting no relation between volume or dose and duration of analgesia. On the other hand, several others reported a direct relation between dose and duration, [7, 11, 12] although in these studies the higher doses were associated with higher volumes as well. Therefore it is unclear whether the effect can be attributed to dose, volume or a combination. In a study using multivariate Cox regression to assess the effect of different volumes and concentrations of ropivacaine on the duration of analgesia following interscalene block for shoulder surgery, Fredrickson et al. [13] concluded that both volume and concentration affect duration independently.

**Fig. 3:**
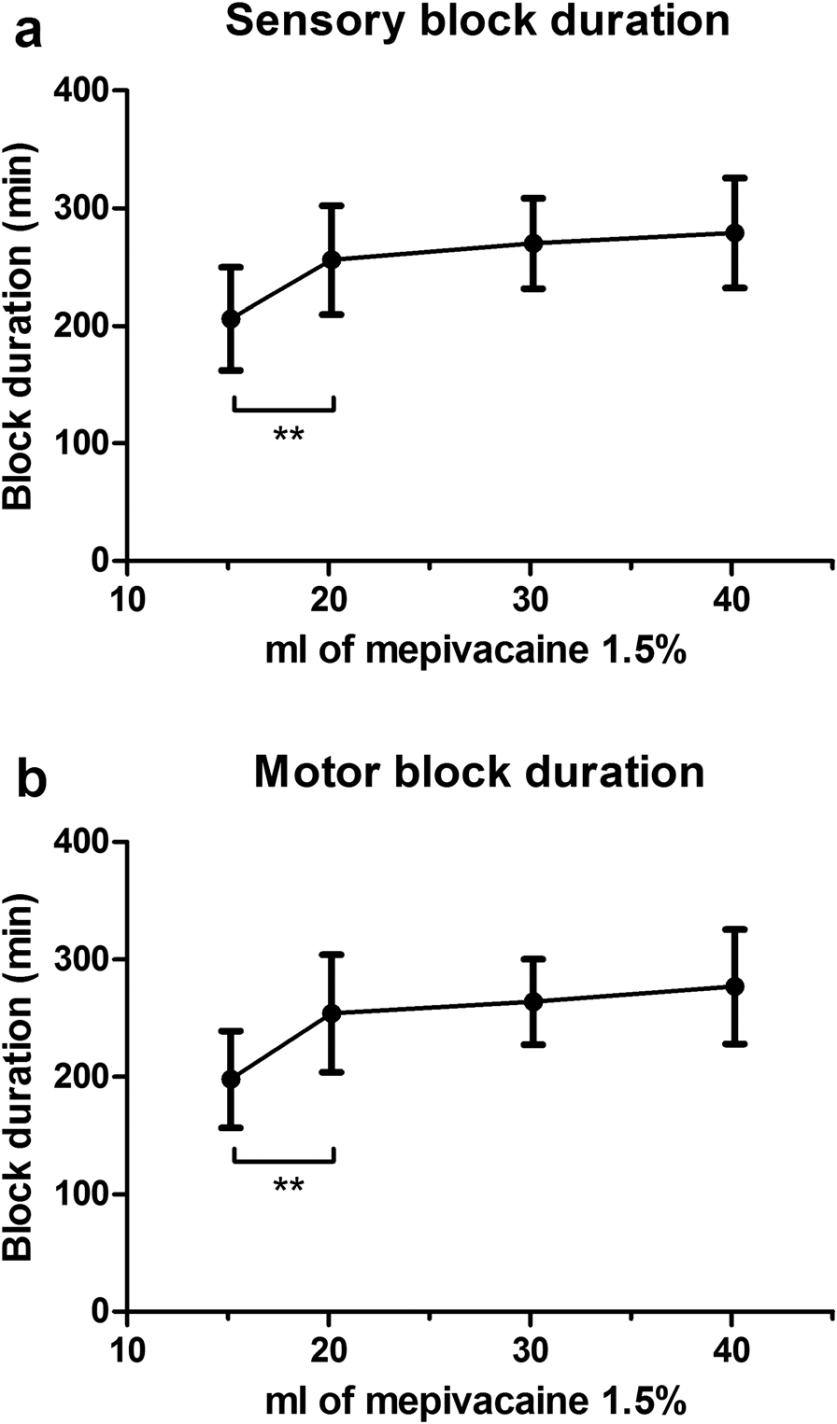
Combined data on sensory block duration (**a**) and motor block duration (**b**) of the present study and 13 patients receiving 15 mL and 15 patients receiving 40 mL mepivacaine 1.5 % from previously published work [7]. Data are presented as mean with SD. *p < 0.005

In a previous study [7] comparing 40 mL and 15 mL mepivacaine 1.5 % for ABPB, we reported that the volume/dose reduction of 62.5 % resulted in a shorter overall duration of sensory and motor block of respectively 17 % and 19 %. In the present study we found that a dose reduction of 33 % did not result in a reduction of block duration (Group A versus Group C). Although comparing results from different studies should be done with caution, the methodology of our present and previous study [7] is identical. Combining the observations from both studies, it seems that the relation between volume/dose and the duration of nervous blockade is not linear. Reducing the volume/dose of mepivacaine 1.5 % from 600 mg (40 mL) to 300 mg (20 mL) results in a modest change in the median duration of nervous blockade of approximately 5 %; a further decrease to 225 mg (15 mL) results in a decrease in duration of approximately 18 % (Fig. 3). It seems therefore that in ABPB with mepivacaine 1.5 %, the optimal balance between volume/dose reduction without significantly affecting duration of nervous blockade is 20 mL.

Three patients were excluded from the study because of block failure, all in Group B. While this may be due to the lower concentration of mepivacaine, our study was not set up nor powered to assess success rate of the different concentrations. In addition, from a clinical perspective, 1 % mepivacaine may not be a suitable choice for ABPB, given the observed failure rate, the inferior onset characteristics, and the shorter duration.

In the patients randomized to group C we exceeded the maximum recommended dose of 4.5 mg/kg mepivacaine. Maximum recommended doses of local anesthetics are usually provided by the manufacturer with the obvious purpose of minimizing the incidence of systemic toxicity, but that does not mean that these recommendations are tantamount to safety. On the contrary, maximum recommended doses are controversial because they are neither evidence based nor specific for site of injection or type of block [14, 15]. In clinical practice larger doses are frequently used and it is our experience that 450 mg mepivacaine for axillary block in adult patients is well within the margin of safety.

A limitation of our study is that we were not able to collect postoperative data of all nerves in all patients because of the presence of a cast or a compression bandage. However, there were no significant differences in the duration of sensory and motor block between the different nerves within each group, with the exception of motor block duration of the musculocutaneous nerve in Group C, and we therefore think that the effect of the missing data on the conclusion of our study is limited.

A second limitation is that our power analysis was based on a clinically relevant difference of 60 min, whereas in retrospect and from a scientific perspective smaller differences may also be interesting. The difference in duration between groups A and B is not statistically significant, but intriguing nevertheless and possibly a larger sample size would have unveiled a significant difference. Future research will focus on further investigating the effect of local anesthetic concentration on duration of sensory and motor block.

## Conclusions

In conclusion, a decrease in volume from 30 to 20 mL mepivacaine does not influence block duration, but a higher dose and concentration in equal volumes of 30 mL results in a longer duration of sensory and motor block in ABPB.
